# Changes in the composition of gut and vaginal microbiota in patients with postmenopausal osteoporosis

**DOI:** 10.3389/fimmu.2022.930244

**Published:** 2022-08-12

**Authors:** Xueli Yang, Tian Chang, Qian Yuan, Wei Wei, Pingping Wang, Xiaojian Song, Huijuan Yuan

**Affiliations:** ^1^ Department of Endocrinology of Henan Provincial People’s Hospital, Henan Provincial Key Laboratory of Intestinal Microecology and Diabetes Control, People’s Hospital of Zhengzhou University, Henan Provincial People’s Hospital of Henan University, Zhengzhou, China; ^2^ Department of Medical and Health, Zhengzhou University Press, Zhengzhou, China

**Keywords:** postmenopausal osteoporosis, gut microbiota, vaginal microbiota, bone mineral density, inflammation

## Abstract

**Background:**

Postmenopausal osteoporosis (PMO) is influenced by estrogen metabolism and immune response, which are modulated by several factors including the microbiome and inflammation. Therefore, there is increasing interest in understanding the role of microbiota in PMO.

**Objectives:**

To investigate variations in gut microbiota (GM) and vaginal microbiota (VM) in postmenopausal women with osteoporosis.

**Methods:**

A total of 132 postmenopausal women were recruited for the study and divided into osteoporosis (n = 34), osteopenia (n = 47), and control (n = 51) groups based on their T score. The serum levels of interleukin (IL)-10, tumor necrosis factor (TNF)-α, and lipopolysaccharide-binding protein were determined *via* enzyme-linked immunosorbent assay. Additionally, 16S rRNA gene V3-V4 region sequencing was performed to investigate the GM and VM of the participants.

**Results:**

Significant differences were observed in the microbial compositions of fecal and vaginal samples between groups (*p* < 0.05). It was noted that for GM, *Romboutsia, unclassified_Mollicutes*, and *Weissella* spp. were enriched in the control group, whereas the abundances of *Fusicatenibacter*, *Lachnoclostridium*, and *Megamonas* spp. were higher in the osteoporosis group than in the other groups. Additionally, for VM, *Lactobacillus* was enriched in the control group, whereas the abundances of *Peptoniphilus, Propionimicrobium*, and *Gallicola* spp. were higher in the osteoporosis group than in the other groups. The predicted functional capacities of GM and VM were different in the various groups. We also found that the serum level of IL-10 in the osteoporosis group was significantly lower than that in the control group and osteopenia group, while TNF-α was significantly higher in the osteoporosis group than that in the control group (*p* < 0.05).

**Conclusion:**

The results show that changes in BMD in postmenopausal women are associated with the changes in GM and VM; however, changes in GM are more closely correlated with PMO than VM.

## 1 Introduction

Postmenopausal osteoporosis (PMO) is a metabolic bone disease mainly caused by estrogen deficiency. It is often characterized by bone loss and bones that fracture easily, among other risks (1, 2). Approximately 10% of the world’s population and over 30% of postmenopausal women aged over 50 years suffer from osteoporosis (3–5). PMO is associated with serious complications, including increased risk of fracture and mortality and has a strong negative impact on mental health (2, 6, 7); therefore, many efforts have been devoted to its prophylaxis and treatment. Remarkable progress has been made in understanding how estrogen deficiency directly results in bone loss; however, the mechanisms involved are complex and multifaceted (3, 7). It has been shown that other factors contribute to bone loss; and, the focus has been on immune response levels, reactive oxygen species, and inflammation, among others (8, 9).

Microorganisms live on the body as well as in the body cavities of humans and communicate with the outside world. The gut microbiota (GM) is a collection of microbes that colonize the human gut and is comprised of about 10 trillion bacteria. The total number of genes in the microbiome is 150 times greater than the total number of genes in human cells (10). The vaginal microbiota (VM) is commonly dominated by *Lactobacillus* spp. These bacteria protect the vagina against colonization by pathogenic bacteria through the production of lactic acid, which lowers vaginal pH. They also produce antimicrobial compounds and modulate the immunological and physical properties of the cervicovaginal mucosa (11). It has been recently hypothesized that the abundance of *Lactobacillus* spp. in the human VM can be influenced by estrogen (12–14). After menopause, the VM is composed primarily of anaerobic and aerobic bacteria (15). The causative role of GM and VM in the development of metabolic diseases such as diabetes and obesity has been established (16). Additionally, the GM plays an important role in osteoporosis (1).

It has been reported that GM may play a role in senile osteoporosis through the “gut microbiome-bone” axis by acting on the immune system, and that the underlying mechanisms involve inflammatory response (1, 17, 18). Consequently, changes in GM may serve as biomarkers for people at high risk of developing senile osteoporosis, whereas microbes in the GM can be used as therapeutic targets (19, 20) Recent animal studies have revealed that the sex steroid hormone-microbio-inflammatory axis could regulate other metabolic disease (21). Moreover, both the GM and VM can affect the inflammatory system, and their metabolic products can trigger local immunological responses with systemic implications (22). The crosstalk of bacterial strains between the gut and vagina stimulates both local and systemic immune responses with attendant effects on the overall host physiology (23). Following the discovery of the influence of GM on senile osteoporosis, it has been recently shown that GM also play a role in PMO (24). However, the role of VM in PMO and the relationship among VM, GM, and PMO are unknown.

In this study, we used 16S rRNA gene V3-V4 region sequencing to investigate the composition and function of GM and VM in postmenopausal women with different bone mineral densities (BMDs). The effects of microbiota on PMO were also studied by evaluating systemic inflammatory response and performing correlation analysis. Our findings suggest that the GM is more closely related to osteoporosis than VM is in postmenopausal women, and that GM other than VM may be a new target in the prevention and treatment of PMO.

## 2 Methods and materials

### 2.1 Participants

This was a cross-sectional study involving patients who were local residents in Henan province and were admitted to the Henan Provincial People’s Hospital (Zhengzhou, China) from August 2019 to October 2020. The participants were matched for age, place of birth, amount of exercise, and eating habits. A total of 132 postmenopausal women were recruited for the study based on inclusion and exclusion criteria; they included 34 patients with osteoporosis, 47 patients with osteopenia, and 51 patients as control group (**Supplementary Figure S1**). This trial was registered in the Chinese Clinical Trial Registry (ChiCTR2000029237) and was approved by the Medical Ethics Committee of Henan Provincial People’s Hospital. Written informed consent was obtained from all participants.

Women who were 45-70 years old and had gone through menopause for more than one year were eligible for inclusion in the study. Patients were excluded from the study if they had fractures as a result of violence or trauma; were suffering from other bone diseases, such as osteomalacia, renal osteodystrophy, or other metabolic bone diseases or bone tumors; had acute or chronic inflammatory or infectious diseases, or were being treated with antibiotics, probiotics, or any medication that could affect intestinal microbiota within the first three months of inclusion; had taken drugs that can cause osteoporosis, such as antidepressants; had serious organic diseases, such as cancer, coronary heart disease, myocardial infarction, or stroke; drank excessively (five or more drinks on the same occasion on each of 5 or more days in the past 30 days (25)); had anemia (hemoglobin level, <10 g/dL); had a physical or self-care disability or were unable to recall clearly and answer questions due to any reason; or did not have time to take part in the project.

### 2.2 Study population and sample collection

The BMDs (g/cm (2)) of the lumbar spine (L1-4) and total hip joint of each participant were measured using a dual-energy X-ray absorptiometry (DXA) scanner (Lunar Expert 1313; Lunar Corp., Madison, WI, USA). The participants were divided into the following groups based on the diagnostic criteria recommended by the World Health Organization (26) and the results of the DXA measurement: T score ≥ -1, control group; -2.5 < T score < -1, postmenopausal osteopenia group; and T score ≤ -2.5, postmenopausal osteoporosis group. Liver function (including alanine transaminase and aspartate transaminase) and kidney function (including creatinine and urea nitrogen) were measured by photometry (Abbott C1600, Illinois, USA). Hematology tests were measured using a hematology Analyzer (Sysmex XN9100, Kobe, Japan), and bone markers were detected by electrochemiluminescence (Cobas E 602, Mannheim, Germany). And gynecological examination was tested by Vaginitis Multitest (Autubio, Zhengzhou, China). Additionally, case report forms requiring the following details were completed for each participant: age, menopausal age, native place, living habits, height, weight, waist-hip ratio (WHR), blood pressure, medical history and medication history, among others. Fasting blood samples, morning urine, feces, and vaginal secretions were collected for analysis. For each participant, fasting blood glucose level was measured when feces and vaginal secretions were collected.

Sterile spoons were used to collect fresh feces in the morning into 8 mL sterilized freezer tubes, after which the samples were immediately stored at -80°C. Contamination of fecal samples with urine was avoided during sample collection. Participants were required to not have sex 24 h before vaginal secretions were taken. During the sample collection, a swab was inserted into the posterior fornix of the vagina, about 5 cm deep from the vaginal opening, and gently rubbed for 10-30 s to avoid contact with the vaginal opening and vulva. The swab was immediately removed, placed in a test tube, and stored at -80°C.

### 2.3 DNA extraction and 16S rRNA gene V3-V4 region sequencing

Genomic DNA was extracted from feces and vaginal swabs using a QIAamp PowerFecal Pro DNA Kit (51804; QIAGEN, Germantown, MD, USA), as in our previous study (27, 28). The composition of the bacterial community in fecal samples was characterized *via* 16S rRNA gene amplicon sequencing. Polymerase chain reaction targeting of the V3-V4 region of the 16S rRNA gene was performed with the following primers: forward, 5’-CCTACGGGNGGCWGCAG-3’; and reverse, 5’-GACTACHVGGGTATCTAATCC-3’. Subsequent amplicon sequencing was performed on a MiSeq platform to generate paired-end reads of 300 bp (Illumina, San Diego, CA, USA).

### 2.4 Sequencing data analysis

VM and GM were analyzed separately. Sequences were analyzed using QIIME2 version 2019.7. Adapters of original sequences were first removed using the “cutadapt” plugin of QIIME2. Sequences were then truncated with DADA2 and further filtered and denoised, after which chimeras were removed. Next, the sequences were merged to obtain the abundance and representative sequences of amplicon sequence variants (ASVs). Representative sequences for ASVs were built into a phylogenetic tree using core-metrics-phylogenetic pipeline in QIIME2, after which taxonomy was assigned using the SILVA database (release 132). All samples for VM and GM analysis were randomly subsampled to equal depths of 12701 and 5103 reads, respectively, prior to fecal microbiome analysis using QIIME2 diversity plugins.

Analysis of α-diversity was performed by using Shannon index, the number of observed ASVs, and Pielou index to evaluate diversity, richness, and evenness, respectively. In contrast, the following tests were used to determine the significance of differences in microbiota structures among the groups for the analysis of β-diversity: principal coordinate analysis, partial least squares discrimination analysis (PLS-DA), and permutational multivariate analysis of variance test (PERMANOVA, 999 tests). PLS-DA was performed and visualized using the R package mixOmics. GraphPad Prism 8.0.1 (GraphPad Software, San Diego, CA, USA) was used to visualize the relative abundances of different bacterial genera in the three groups. Linear discriminant analysis effect size (LEfSe) (http://huttenhower.sph.harvard.edu/galaxy/) was used to identify key differences in ASVs between the control, osteopenia, and osteoporosis groups. The heatmap for key ASVs was visualized using MATLAB 2019b (The MathWorks, Inc., Natick, MA, USA).

### 2.5 Correlation analysis

MATLAB R2019b was used to calculate Spearman correlation coefficients between key ASVs and clinical parameters. The Benjamini and Hochberg method was used to calculate false discovery rate (FDR) to adjust the significance of correlations. The network of correlation results was generated using Cytoscape v3.7.2 (https://cytoscape.org/).

### 2.6 Prediction of Kyoto encyclopedia of genes and genomes metabolic pathways

PICRUSt2 was used to predict the metabolic functions of VM and GM based on the representative sequences obtained from using QIIME2. The different metabolic pathways between the three groups of participants were selected using LEfSe, whereas MATLAB R2019b was used to generate a heatmap for different KEGG pathways.

### 2.7 Enzyme-linked immunosorbent assay

The serum levels of interleukin (IL)-10, tumor necrosis factor (TNF)-α, and lipopolysaccharide-binding protein (LBP) were determined using commercially available ELISA kits (Cusabio Biotech, Wuhan, China) according to the manufacturer’s instructions.

### 2.8 Statistical tests

#### 2.8.1 Statistical analysis of clinical data

Statistical analysis of clinical data was performed using Statistical Package for the Social Sciences (version 22; IBM Corp., Armonk, NY, USA). Data have been expressed as the mean or median with interquartile range based on whether the data fit the normal distribution. All biochemical parameters were analyzed using one-way analysis of variance or nonparametric Kruskal–Wallis test.

#### 2.8.2 Statistical analysis of GM and VM data

Kruskal–Wallis test with Dunn’s multiple tests was used to test differences in α-diversity indexes among the three groups using GraphPad Prism 8.0.1. The same test was used to evaluate differences in unweighted UniFrac distances between the microbiota of women in the different groups. Wilcoxon rank-sum test was used to investigate differences in the abundance of key ASVs between groups using the R package MASS. *P*-values were adjusted to the FDRs using the original FDR method of Benjamini and Hochberg.

## 3 Results

### 3.1 Analysis of clinical characteristics

The study population was divided into control, osteopenia, and osteoporosis groups according to the T values obtained. Compared with the control group, BMD decreased gradually in the osteopenia and osteoporosis groups (*p <* 0.001). Furthermore, compared with the control group, age and time after menopause increased in the osteopenia group, body mass index (BMI) and estrogen levels decreased in the osteoporosis group (*p <* 0.05) (**Supplementary Table S1**). The age of each group tended to increase gradually with severer disease. No significant differences were observed in WHR or the levels of alanine transaminase, aspartate transaminase, total cholesterol, triglyceride, high-density lipoprotein, low-density lipoprotein, and bone markers (25-hydroxyvitamin D_3_ [25(OH)VD_3_], osteocalcin, procollagen type 1 N-terminal propeptide, β-Crosslaps) among the three groups (**Supplementary Table S1**).

### 3.2 Significant differences in GM structure and composition among the groups

**Figure 1:**
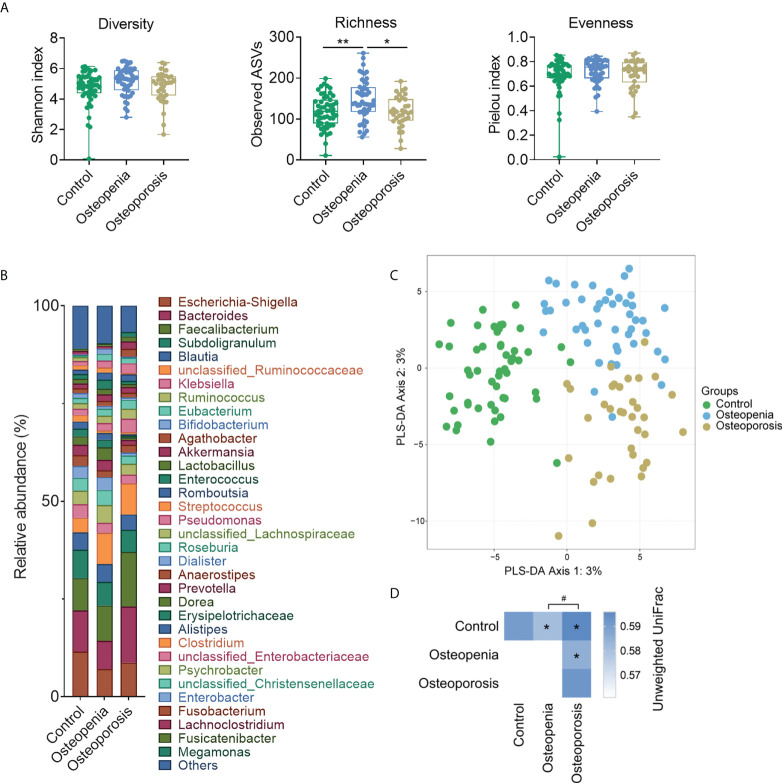
Structure and composition of GM among the control, osteopenia, and osteoporosis groups. **(A)** Diversity, richness, and evenness of GM in the groups. **(B)** Bar plot showing the relative abundance of each genus. Only genera whose average relative abundances are greater than 1% among the samples are shown. **(C)** PLS-DA plot. **(D)** PERMANOVA test results based on the unweighted UniFrac distance of GM and comparison of unweighted UniFrac distances between the microbiota of women in the osteopenia, osteoporosis, and control groups. * and ** represent PERMANOVA *p*-values <0.05 and <0.01, respectively. ^#^ represents *p* < 0.05 from the Wilcoxon rank-sum test of the distances.

Our results did not show any difference in the diversity and evenness of GM among the three groups (**Figure 1A**). However, GM richness was significantly higher in the osteopenia group than in the control or osteoporosis group (**Figure 1A**). Moreover, compared with the control group, *Bacteroides* and *Bifidobacterium* spp. showed an increasing trend in relative abundance in the osteoporosis group, whereas *unclassified_Ruminococcaceae* showed a decreasing trend in the osteopenia and osteoporosis groups (**Figure 1B**). Unweighted UniFrac distance was used to evaluate the β-diversity of GM. And, the structure of GM of three groups can be separated notably by PLS-DA and PCoA (**Figure 1C** and **Supplementary Figure S2**). The PERMANOVA test results based on unweighted UniFrac distance also showed that the structure among control, osteopenia and osteoporosis groups were significant (**Figure 1D**). After further adjustment of time after menopause and BMI separately, all the between-group differences of structure in GM remained significant. While age was taken as a covariable, only the difference between the control group and osteopenia group became insignificant (*p* = 0.060). And, as estrogen was a covariable, the differences of osteopenia group versus control group (*p* = 0.077) and osteoporosis group versus control group (*p* = 0.064) disappeared. In addition, the difference between control group and osteoporosis group (*p* = 0.123) disappeared when time after menopause, age, estrogen and BMI were taken as covariables together, while the differences of control group versus osteopenia group (*p* = 0.640) and control group versus osteoporosis group (*p* = 0.086) were both disappeared when time after menopause, BMI and estrogen were taken as covariables together. (**Supplementary Table S2**).

### 3.3 Different genera and KEGG pathways of GM among the three groups

**Figure 2:**
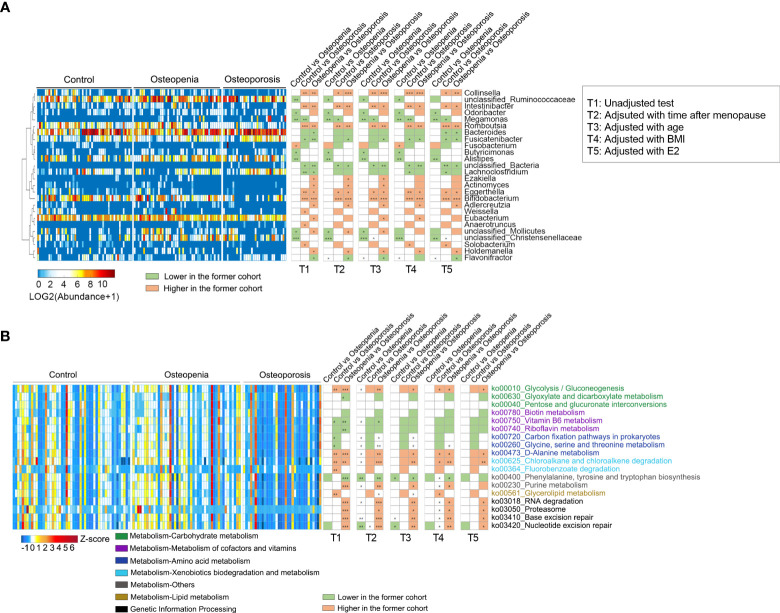
Different genera and KEGG pathways of GM among the control, osteopenia, and osteoporosis groups. **(A)** Different genera in the GM. The cluster of genera based on Spearman correlation coefficients is shown on the left. The heatmap in the middle shows the relative abundance (log2 transformed) of each genus in individual samples. Comparisons of the genera are shown in the right columns of the heatmap. All the genera were tested using the Wilcoxon rank-sum test and adjusted to FDRs.*, ** and *** represent FDR < 0.05, < 0.01 and < 0.01, respectively. **(B)** Differential predicted KEGG pathways. The heatmap in the middle shows the relative abundance (Z-score) of pathways in individual samples. All the pathways were tested using Wilcoxon rank-sum test and adjusted to FDRs. *, ** and *** represent FDR < 0.05, < 0.01 and < 0.01, respectively.

We analyzed the relative abundance of the detected genera of GM among the three groups (**Supplementary Table S8**). It was found that compared with the control group, the abundances of eight bacterial genera in the osteopenia group changed significantly. For example, the abundance of *Megamonas* spp., which are associated with inflammation (29), was significantly increased. Furthermore, the abundances of 12 bacterial genera in the osteoporosis group changed significantly. For example, the abundances of *Megamonas*, *Lachnoclostridium*, and *Fusicatenibacter* spp. were significantly higher, whereas those of *Collinsella* [which can produce butyrate (30)], *Romboutsia*, and *Bifidobacterium* spp. were significantly lower in the osteoporosis group than in the control group (**Figure 2A** and **Supplementary Figure S3**). Time after menopause, age, BMI and estrogen were further adjusted respectively, few between-group differences in the abundance of GM (mainly between the control group and osteoporosis) disappeared, such as *Weissella*, while most between-up differences in the abundance of GM remained significant, such as *Megamonas*, *Collinsella, Romboutsia*, and *Bifidobacterium* spp. However, when time after menopause, estrogen and BMI were taken as covariables together, the between-group differences were significantly reduced. Especially when age was also included as a covariable altoghter, only individual genus such as *Lachnoclostridium* spp. showed between-group differences (**Figure 2A** and **Supplementary Table S6A**).

PICRUSt2 was used to predict the metabolic function of GM, and 18 key metabolic pathways were found. It was noted that compared with the control group, pathways associated with amino acid synthesis and genetic information processing increased in the osteopenia group, whereas those related to carbohydrate metabolism and lipid metabolism significantly decreased in the osteoporosis group. In addition, purine metabolism and genetic information processing pathways were significantly lower in the osteoporosis group than in the osteopenia group (**Figure 2B**). Further, time after menopause, age, estrogen and BMI were used as covariables respectively, the between-group differences of most metabolic pathways are similar, especially for genetic information processing. Nonetheless, when these covariables were analyzed together, the between-group differences of metabolic pathways decreased significantly, which were similar to the change in genus level (**Figure 2B** and **Supplementary Table S6B**).

### 3.4 Significant differences in VM structure and composition among the groups

**Figure 3:**
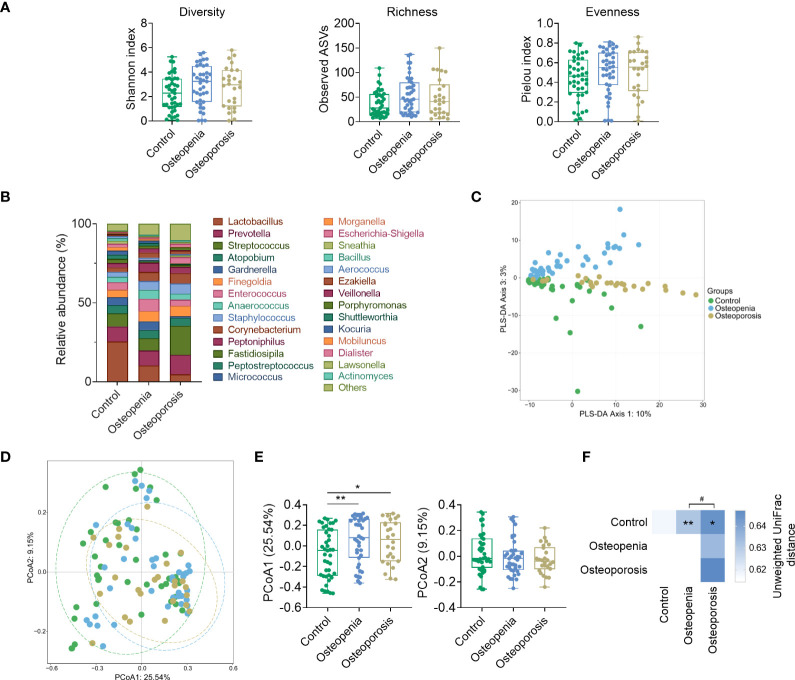
Structure and composition of VM among the control, osteopenia, and osteoporosis groups. **(A)** Diversity, richness, and evenness of VM in the groups. **(B)** Bar plot showing the relative abundance of each genus in the VM. Only genera whose average relative abundances are greater than 1% among the samples are shown. **(C)** PLS-DA plot. **(D)** Principal component analysis plot based on unweighted UniFrac distance. **(E)** Comparisons of sites in principal components 1 and 2. **(F)** PERMANOVA test result based on unweighted UniFrac distance of VM and the comparison of unweighted UniFrac distances between the microbiota of women in the osteopenia, osteoporosis, and control groups. * and ** represent PERMANOVA *p*-values < 0.05 and < 0.01, respectively. ^#^ represents *p* < 0.05 from the Wilcoxon rank-sum test of the distances.

VM specimens were not collected from 26 patients. Our analysis showed no significant difference in the α-diversity of VM among the control, osteopenia, and osteoporosis groups (**Figure 3A**). However, differences were observed in the compositions of some bacterial genera. For instance, compared with the control group, *Lactobacillus* spp. significantly decreased in the osteopenia and osteoporosis groups, whereas *Streptococcus* spp. significantly increased in the osteoporosis group (**Figure 3B**). And, the overall structure of VM of three groups can be separated notably by PLS-DA and PCoA (**Figures 3C–E**). PERMANOVA analysis based on the unweighted UniFrac distances revealed that the structure of VM in the osteopenia and osteoporosis groups was significantly different from that in the control group (**Figure 3F**). After taking time after menopause, age and estrogen as covariates respectively, the differences of structure in VM between the control group and the osteoporosis group became not statistically significant. While these covariables were analyzed together, the differences of structure in VM were similar to those without correction (**Supplementary Table S3**).

### 3.5 Different genera and KEGG pathways of VM among the three groups

**Figure 4:**
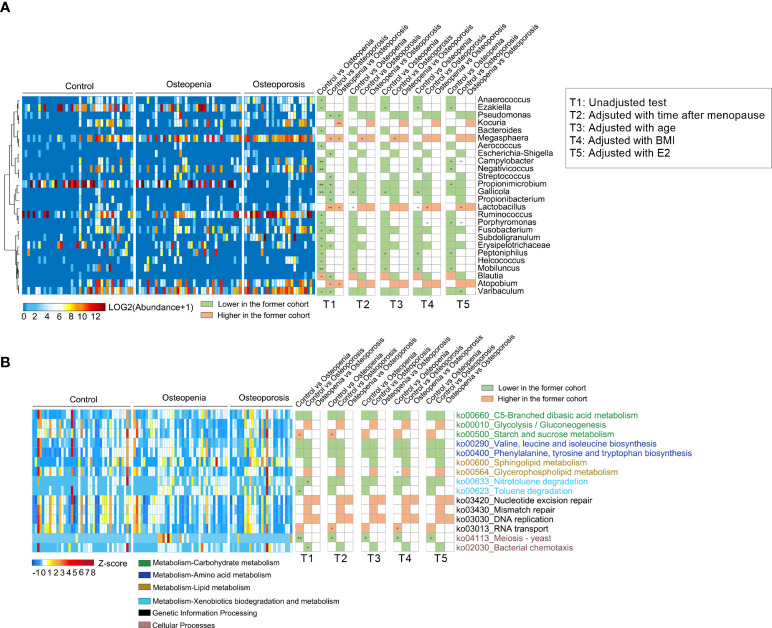
Different genera and KEGG pathways of VM among the control, osteopenia, and osteoporosis groups. **(A)** Different genera in the VM. The cluster of genera based on Spearman correlation coefficients is shown on the left. The heatmap in the middle shows the relative abundance (log2 transformed) of each genus in individual samples. Comparisons of the genera are shown in the right columns of the heatmap. All the genera were tested using Wilcoxon rank-sum test and adjusted to FDRs. * and ** represent FDR < 0.05 and < 0.01, respectively. **(B)** Differential predicted KEGG pathways. The heatmap in the middle shows the relative abundance (Z-score) of pathways in individual samples. All the pathways were tested using Wilcoxon rank-sum test and adjusted to FDRs. * and ** represent FDR < 0.05 and < 0.01, respectively.

We analyzed the relative abundance of the detected genera of VM among the three groups (**Supplementary Table S9**). The abundances of 18 bacterial genera changed significantly in the osteopenia group; however, this was not observed in the control group. Specifically, the abundances of *Peptoniphilus* and *Anaerococcus* spp. and other bacteria significantly increased, whereas the abundance of *Blautia* spp. significantly decreased. Moreover, the abundances of 13 bacterial genera in the osteoporosis group changed significantly. The results showed that the abundances of *Streptococcus* spp., which are significantly associated with inflammatory disease (31), and *Fusobacterium* spp., which are opportunistic pathogens (32), were significantly higher, whereas those of *Lactobacillus*, *Atopobium*, and *Megasphaera* spp. were significantly lower in the osteoporosis group than in the control group (**Figure 4A** and **Supplementary Figure S4**). However, in a covariate analysis of time after menopause, most of the between-group differences of VM disappeared, such as *Fusobacterium, Propionimicrobium, Streptococcus* spp., while the between-group differences of *Gallicola* spp., etc. were still significant. Further, time after menopause, age, estrogen and BMI were taken as covariables, only *Lactobacillus* spp. showed significant differences of control group versus osteopenia group and control group versus osteoporosis group (**Figure 4A** and **Supplementary Table S7A**).

The metabolic function of VM was predicted, and it was found that 15 key metabolic pathways were involved. The results showed that in the osteopenia group than the control group, pathways related to carbohydrate metabolism and amino acid metabolism also increased, whereas starch and sucrose metabolism and RNA transport decreased. Furthermore, in the osteoporosis group, carbohydrate metabolism, lipid (sphingolipid) metabolism, and other pathways increased, whereas glycolysis decreased. Additionally, pathways related to genetic information processing were fewer in the osteoporosis group than in the osteopenia group (**Figure 4B**). After further adjustment of time after menopause, age, estrogen and BMI, individually or together, the between-group differences of metabolic pathways decreased and the residual differences still concentrated on the above-mentioned aspects (**Figure 4B** and **Supplementary Table S7B**).

### 3.6 Changes in systemic inflammation

**Figure 5:**
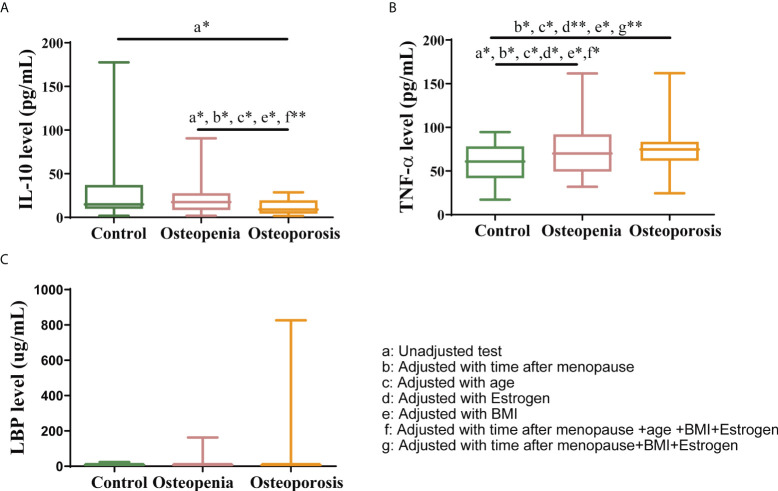
Changes in systemic inflammation status. Serum levels of **(A)** IL-10, **(B)** TNF-α and **(C)** LBP. The data have been presented as a box and whisker plot (vertical). The line in the middle of each box was plotted at the median, whereas the inferior and superior limits of the box correspond to the 25th and 75th percentiles, respectively. The whiskers correspond to the minimum to maximum values. A Kruskal–Wallis test was used to compare the differences among the three groups. Mann-Whitney U test was used to detect the differences among the two groups. All the covariates were adjusted using analysis of covariance (ANCOVA) by R package multcomp. * indicates *p* < 0.05, ** indicates *p* < 0.01.

Previous studies have shown GM and VM regulates inflammation (9, 23, 33–35), which is related to osteoporosis, so we hypothesized that osteoporosis may be associated with an increase in systemic inflammation by GM and VM. The results in this study showed that the level of the anti-inflammatory factor IL-10 was significantly lower in the osteoporosis group than in the control and osteopenia groups (**Figure 5A**). Moreover, the level of the inflammatory factor TNF-α was significantly higher in the osteopenia group than in the control group (**Figure 5B**). There was no statistical difference in LBP level between the groups. LBP can bind to antigens, such as endotoxins, produced by bacteria. Additionally, it is a surrogate biomarker that is indicative of the link between bacterial antigen load in blood and host inflammatory response (36, 37). Our data show that LBP level tended to increase with severer disease (**Figure 5C**). While the levels of interleukin (IL)-33, associated with bone-protecting (24), and interleukin (IL)-17A, associated with bone destruction (9), were not statistically different between groups (**Supplementary Figure 5** and **Supplementary Table S4**). After taking time after menopause, age, estrogen and BMI as covariates separately, the differences of IL-10 disappeared while the differences of TNF-α became significant between the control group and the osteoporosis group. Meanwhile, differences of IL-10 in osteopenia group versus osteoporosis group, and differences of TNF-α in control group versus osteopenia group were still significant. Besides, after taking time after menopause, age, estrogen and BMI as covariates together, the difference of IL-10 between the osteopenia group and the osteoporosis group was still significant. After taking time after menopause, estrogen and BMI as covariates together, the differences of TNF-α in control group versus osteopenia group and control group versus osteoporosis group were significant (**Supplementary Table 5**).

### 3.7 Correlation network between key genera in the gut and vagina, the levels of inflammatory factors, and clinical phenotypes

Spearman correlation analysis was performed to evaluate the correlation between GM, VM, the levels of inflammatory factors, and clinical index values, after which the data were visualized.

*Romboutsia* and *Eggerthella* spp. in the GM level, which significantly decreased with severer disease, were found to have a significantly positive correlation with serum IL-10 level and femoral neck (FN) T-score. Additionally, *Fusicatenibacter* spp., which were significantly increased in the osteoporosis group, were found to have a significantly positive correlation with the time after menopause but a negative correlation with serum IL-10 and estrogen levels. *Lachnoclostridium* had a significantly negative correlation with serum IL-10 and estrogen levels, whereas *Bifidobacterium* spp., which significantly increased in the osteoporosis group, had a significantly positive correlation with lumbar spine BMD (LS BMD).

**Figure 6:**
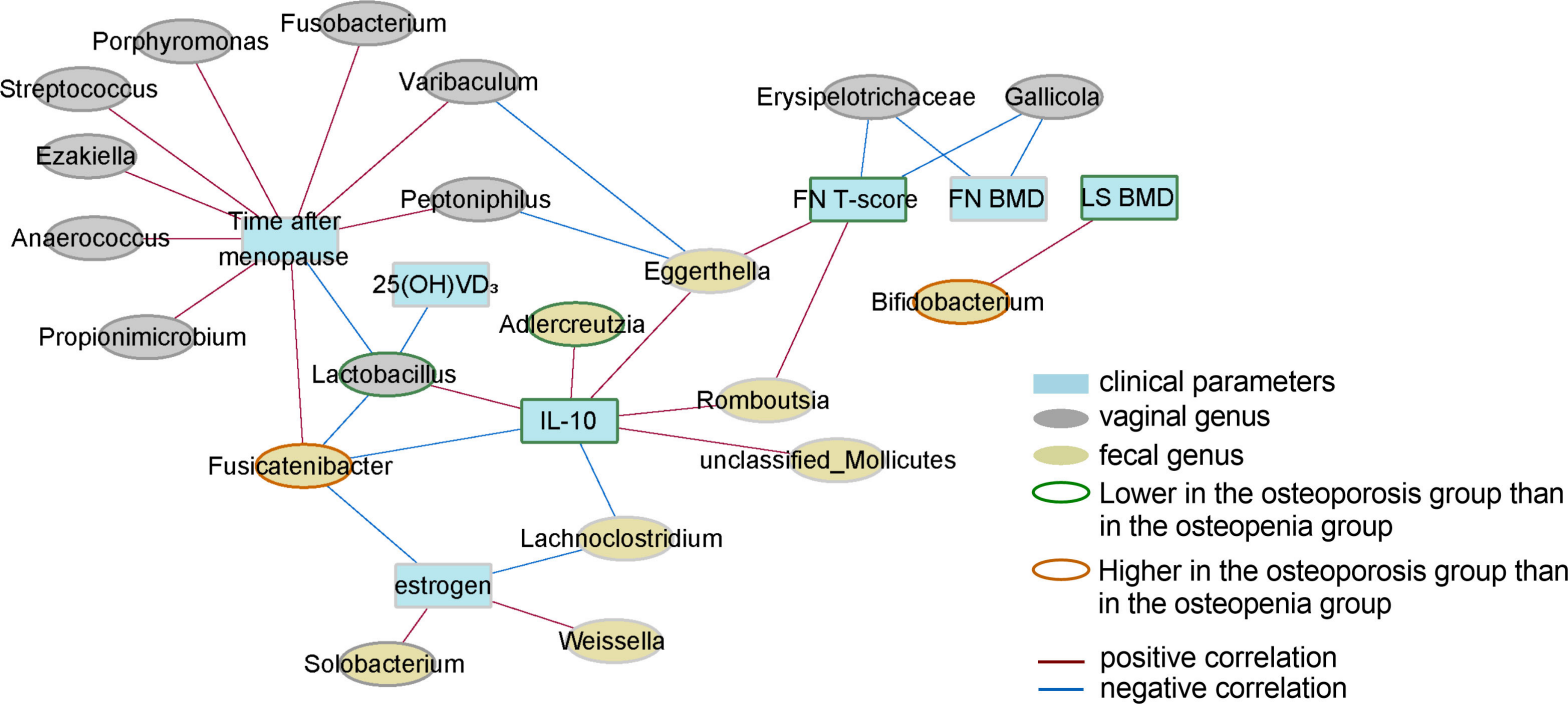
Correlation network of key genera in GM and VM, the levels of inflammatory factors, and clinical phenotypes. Only correlations with FDR values < 0.05 are shown. The width of a line represents the absolute value of the Spearman correlation coefficient. The color of a line represents correlation type; blue represents a negative correlation, whereas red represents a positive correlation. The node border color represents the result of the comparison of genera/parameters between the osteoporosis and osteopenia groups.

With respect to the level of VM, the results showed that *Lactobacillus* had a significantly positive correlation with serum IL-10 level but a negative correlation with time after menopause, 25(OH)VD_3_ level, and intestinal *Fusicatenibacter*. Additionally, *Gallicola* and *Erysipelotrichaceae* were found to have a significantly negative correlation with FN T-score and FN BMD. It was also noted that *Porphyromonas* and *Streptococcus* had a significantly positive correlation with time after menopause (**Figure 6**). The correlation data are shown in **Supplementary Tables S10-12**.

In addition, we calculated the correlation between key predicted pathways of GM and VM and clinical parameters. We found that the proteasome pathway of GM was significantly positively correlated with BMD (**Supplementary Figure S6A**), while the correlation between the pathway of VM and clinical parameters mainly focused on time after menopause (**Supplementary Figure S6B**).

## 4 Disscussion

It has been indicated in numerous reports that alterations in the gut microbiome are associated with changes in bone mass in osteoimmunology and gut microbiome-bone axis theory (38, 39). However, a few studies have been conducted to assess the role of microbiota in PMO, the most common type of osteoporosis (3). Moreover, the relationship between VM, which is closely associated with menopause (16) and inflammation (21), and PMO has not been studied yet. In the present study, we found that postmenopausal women with different BMDs had different GM compositions, which is similar to a previous finding (24). It was also found that VM composition changed significantly with severer disease. Moreover, further correlation and functional analyses of the microbiota revealed that BMD changes may be closely related to GM and VM, metabolic response, and inflammation level, and that these factors appear to form a relationship network.

Our findings suggested that decreases in BMD were associated with changes in GM and VM, which may be closely related to the pathogenesis of osteoporosis. With respect to GM, it was observed that the levels of *Romboutsia* spp., which are predicted to be associated with the production of the short-chain fatty acid acetate and a decreased risk of infection, and *Bifidobacterium* spp., which can increase bone density by promoting the absorption of minerals such as calcium, magnesium, and phosphate (40), decreased significantly in the osteoporosis group. In contrast, the proportion of *Megamonas* spp., which are significantly correlated with systemic inflammatory cytokines (29, 41), significantly increased. With respect to VM, we found that the levels of *Lactobacillus* spp. decreased with severer disease. *Lactobacillus* spp. are the dominant vaginal bacterial species and are beneficial to host health, as they are involved in the regulation of glucose and lipid metabolism and can alleviate oxidative stress and inflammatory response (15, 42). *Streptococcus* spp. can promote inflammation by affecting the metabolic pathways of sphingolipids, pyruvate, and inositol phosphate (31). It was also reported that *Propionimicrobium* spp. can increase the levels of pro-inflammatory cytokines (43). Our results showed that the proportions of *Streptococcus* and *Propionimicrobium* spp. were significantly higher in the osteoporosis group than in the other groups.

Functional analysis of microbiota and analysis of the correlation among the microbiota, clinical indicators, and inflammatory factors were performed to further clarify the results. We initially hypothesized that the mechanism underlying the observed results leads to a potential immune-mediated effect. Undeniably, the association between the GM and bone healing and remodeling is partly moderated by immune cells (44). Moreover, GM and VM can have an effect on inflammation, and their metabolic products can trigger local immunological responses with systemic implications. The results showed that the proportion of bacteria involved in butyric acid production significantly decreased in the osteoporosis and osteopenia groups than the control group. Butyric acid can regulate bone metabolism in various ways (45, 46). It was reported that people with metabolic diseases and inflammatory diseases usually have low levels of butyrate-producing bacteria in their intestines (47), and that inflammatory cytokines can enhance osteoclast activity, leading to a decrease in bone mass (48). Results of the functional analysis indicated that carbohydrate metabolism by GM was significantly decreased, and that complex carbohydrates were absorbed and converted into simple sugars, which were further fermented to produce short-chain fatty acids such as butyric acid (49). And, after further analysis of the correlation between key predicted pathways and clinical parameters, the proteasome pathway significantly reduced in the osteoporosis group was significantly positively correlated with BMD, and could degrade HIF-1 α protein which is associated with bone erosion and expressed in RANKL-stimulated osteoclast precursor cells (50). Furthermore, sphingolipid metabolism by VM, which was enriched in the osteoporosis group than the control group, is known to play a key role in the regulation of inflammation signaling pathways. Additionally, it is reported that dietary sphingolipids can have an effect on inflammatory chronic diseases by inhibiting intestinal lipid absorption, altering GM, activating anti-inflammatory nuclear receptors, and neutralizing responses to inflammatory stimuli (51).

In addition to few microbes in the VM that were related to BMD and IL-10 and estrogen levels, most of the microbes were closely and positively related to time after menopause. In contrast, gut microbes that were associated with BMD and IL-10 and estrogen levels were more abundant. Further covariance analysis showed that after adjusting for the related variables separately, especially for the time after menopause, the between-group differences of VM were significantly reduced, while when the time after menopause, age, estrogen and BMI were taken as covariables together, the between-group differences of GM were reduced. So we suggested that, compared to VM, GM was more closely related to BMD and the other factors. We hypothesized that this was possibly because the distribution of VM was relatively limited compared to that of GM. Additionally, current research on VM is primarily related to female health and reproductive outcomes (conception and birth) (23, 52). Therefore, it is possible that the VM is less important than the GM is with respect to systemic metabolic diseases, which suggests that GM may be more suitable as a new target for PMO management.

One limitation of our study was the small size used. In addition, this study was a cross-sectional study and possibly lacked in-depth mechanism research. In our further experiments, we plan to transplant GM from the control group and the osteoporosis group separately into germ-free rats with postmenopausal osteoporosis. Thus, the changes of osteoporosis-related factors in the transplanted rats can be observed, so as to analyze and verify the causal relationship between GM and the development of postmenopausal osteoporosis. Further validation analysis is to identify the key strains affecting postmenopausal osteoporosis through metagenomic sequencing, and transplant the specific strains into rats with postmenopausal osteoporosis to verify the causal relationship and mechanism on postmenopausal osteoporosis.

## 5 Conclusion

In this study, the profiles and compositions of VM and GM in postmenopausal women with osteopenia and osteoporosis were investigated. We identified distinguishing features of the GM and VM of the participants in the study. Additionally, the functions of GM and VM as well as their relationships with inflammatory factors and metabolic pathways that can influence osteoporosis were studied. Our results provide new insights into the effects of GM and VM on PMO. Specifically, we found that GM is more closely related to PMO than VM is, further suggesting that GM may be more suitable as a new target in the management of PMO.

## Data availability statement

The datasets presented in this study can be found in online repositories. The name of the repository and accession number can be found below: NCBI Sequence Read Archive; SRP372123.

## Ethics statement

The studies involving human participants were reviewed and approved by the Medical Ethics Committee of Henan Provincial People’s Hospital. The patients/participants provided their written informed consent to participate in this study.

## Author contributions

HY, TC and XY designed this study. TC, QY, WW, and PW recruited and supervised the participants and performed all experimental procedures. PW supervised operation of ELISA. XY and XS performed statistical analysis and generated the figures and tables. XY and TC prepared the manuscript prepared the manuscript. All authors contributed to the article and approved the submitted version.

## Funding

This work was financially supported by National Natural Science Foundation of China (81970705); Centaline Thousand Talents Plan-Leading Talents of Science and Technology Innovation in Centaline (204200510026).

## Conflict of interest

The authors declare that the research was conducted in the absence of any commercial or financial relationships that could be construed as a potential conflict of interest.

## Publisher’s note

All claims expressed in this article are solely those of the authors and do not necessarily represent those of their affiliated organizations, or those of the publisher, the editors and the reviewers. Any product that may be evaluated in this article, or claim that may be made by its manufacturer, is not guaranteed or endorsed by the publisher.
