# Supplementary material for: Changes in the composition of gut and vaginal microbiota in patients with postmenopausal osteoporosis
Source: Front Immunol. 2022 Aug 12;13:930244. doi: 10.3389/fimmu.2022.930244 (PMC9411790; doi:10.3389/fimmu.2022.930244)
Supplement: Supplementary file 1 [file Presentation_1.pdf]

Supplementary Material

1 Supplementary Figures

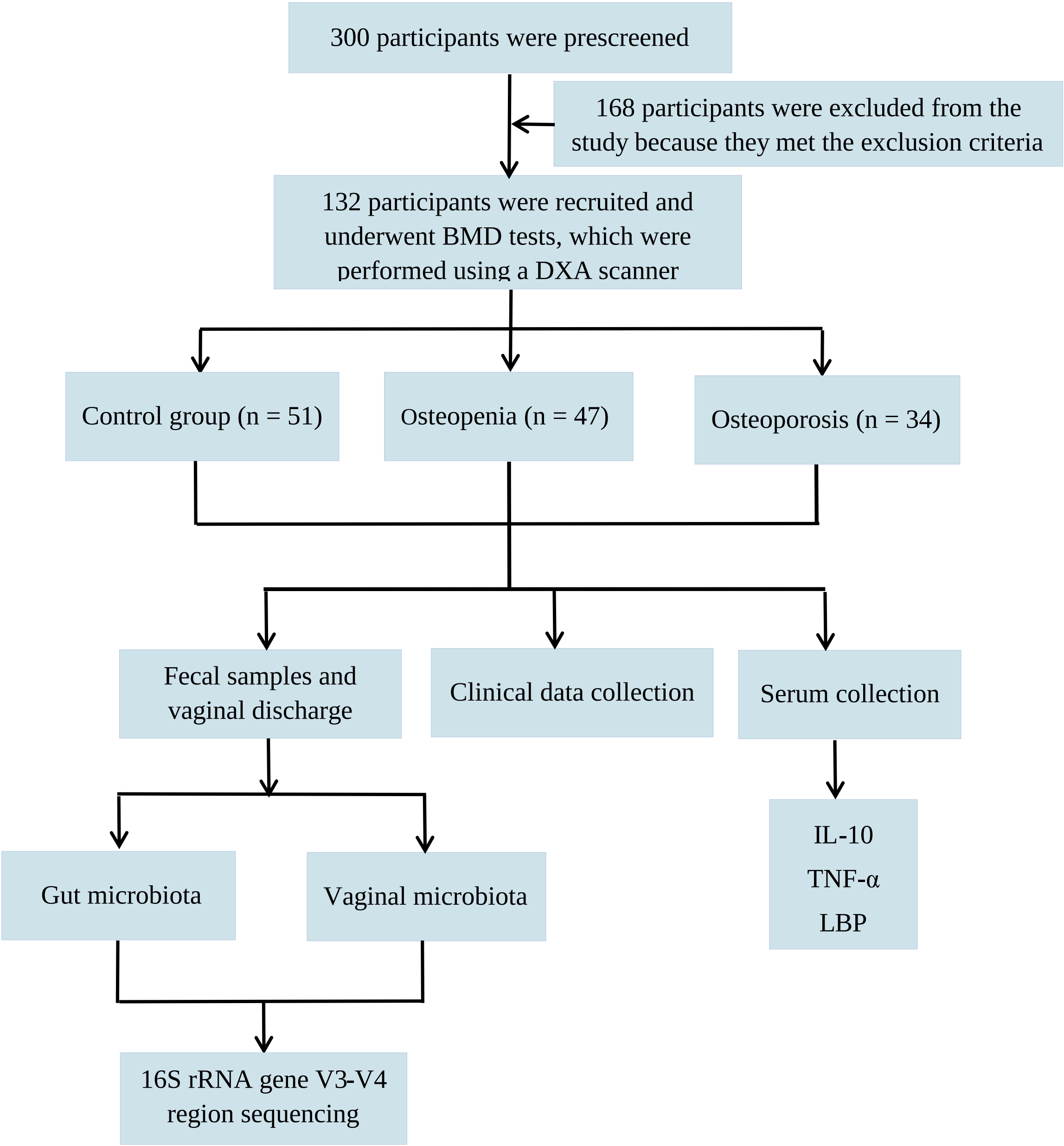

Supplementary Figure 1. Trial profile. Inclusion criteria: aged 45 to 70 years; suspension for more than 1 year. Exclusion criteria: fractures caused by violence or trauma; suffered from other bone diseases, such as osteomalacia, renal osteodystrophy and other metabolic bone diseases or bone tumors; had acute or chronic inflammatory or infectious diseases, or who are being treated with antibiotics, probiotics, probiotics or any other medication that may affect the intestinal microbiota in

the first three months of inclusion; had taken drugs that can cause osteoporosis, such as antidepressant; had serious organic diseases, such as cancer, coronary heart disease, myocardial infarction, or stroke; drank excessively; had anemia (hemoglobin level, <10 g/dL); had a physical disability or self-care disability or were unable to recall clearly and answer questions due to any reason; lacked the time to take part in this project. BMD: bone mineral density; DXA: dual-energy X-ray absorptiometry; IL: interleukin; TNF: tumor necrosis factor; LBP: lipopolysaccharide-binding protein.

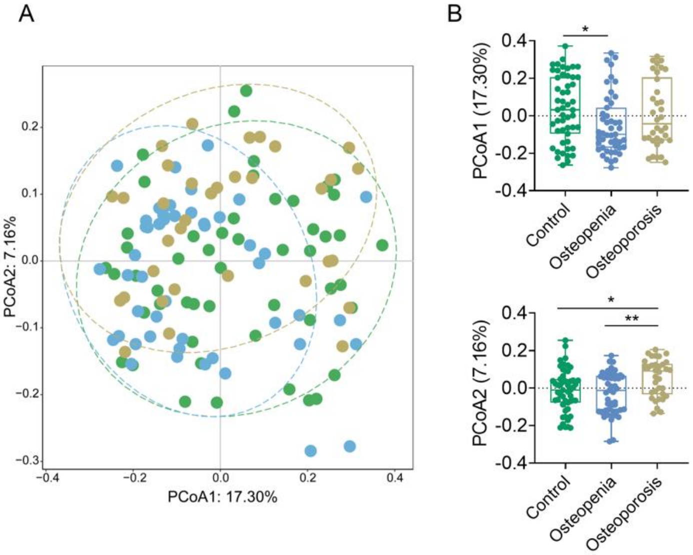

Supplementary Figure 2. Comparison of the overall structure of gut microbiota between groups. **(A)** Principal component analysis plot based on unweighted UniFrac distance. **(B)** Comparisons of the sites in principal components 1 and 2.

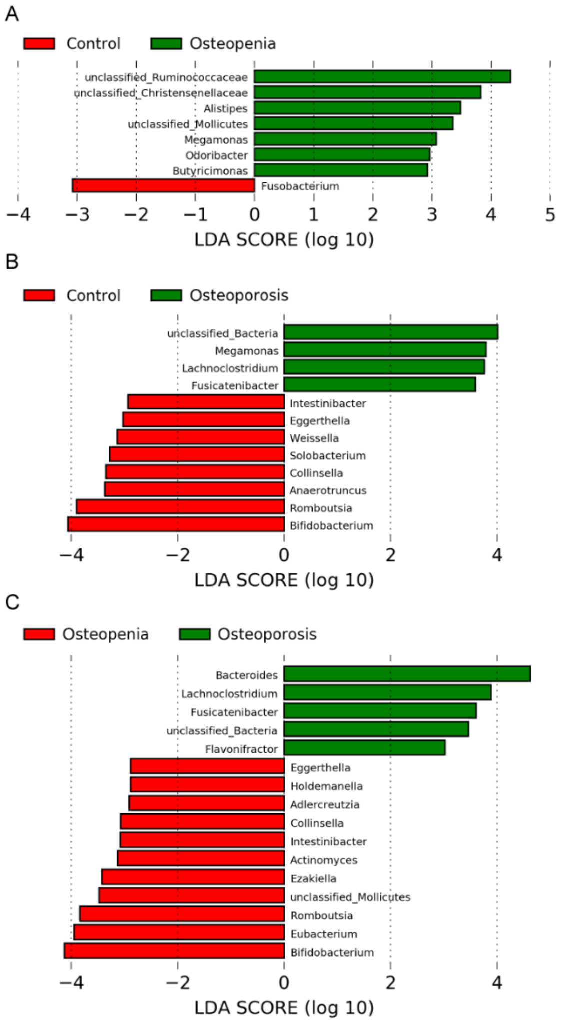

Supplementary Figure 3. Comparison of differences in gut microbiota composition between groups via LEfSe analysis (genus level). **(A)** Control and osteopenia groups. **(B)** Control and osteoporosis groups. **(C)** Osteopenia and osteoporosis groups.

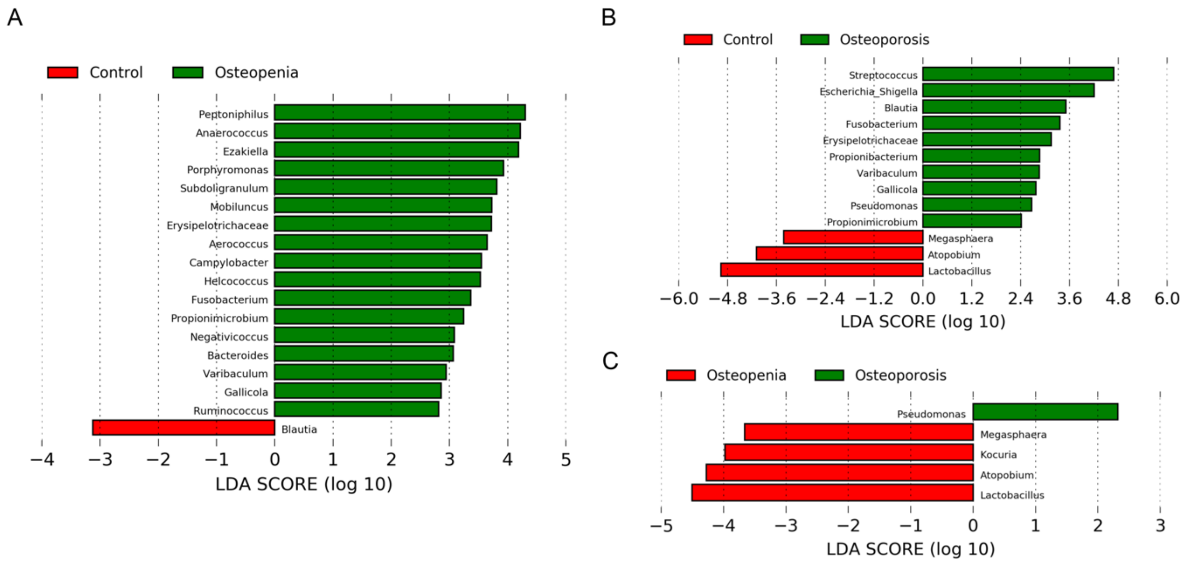

Supplementary Figure 4. Comparison of differences in vaginal microbiota composition between groups via LEfSe analysis (genus level). **(A)** Control and osteopenia groups. **(B)** Control and osteoporosis groups. **(C)** Osteopenia and osteoporosis groups.

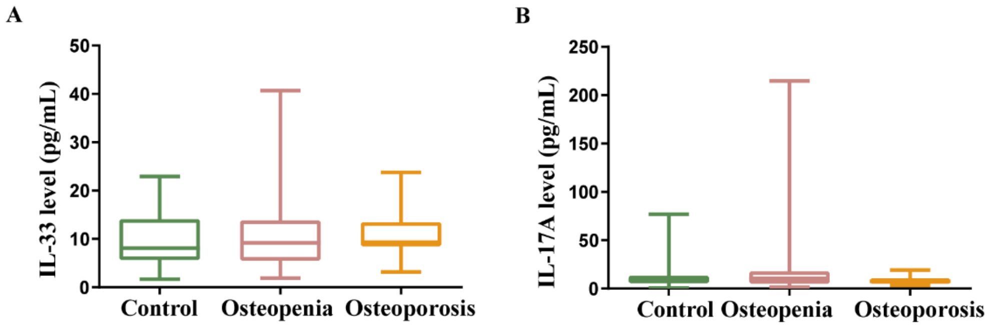

Supplementary Figure 5. Serum levels of IL-33 and IL-17A. The data have been presented as a box and whisker plot (vertical). The line in the middle of each box plot is at the median, whereas the inferior and superior limits of each box correspond to the 25th and 75th percentiles, respectively. The whiskers correspond to minimum to maximum values.

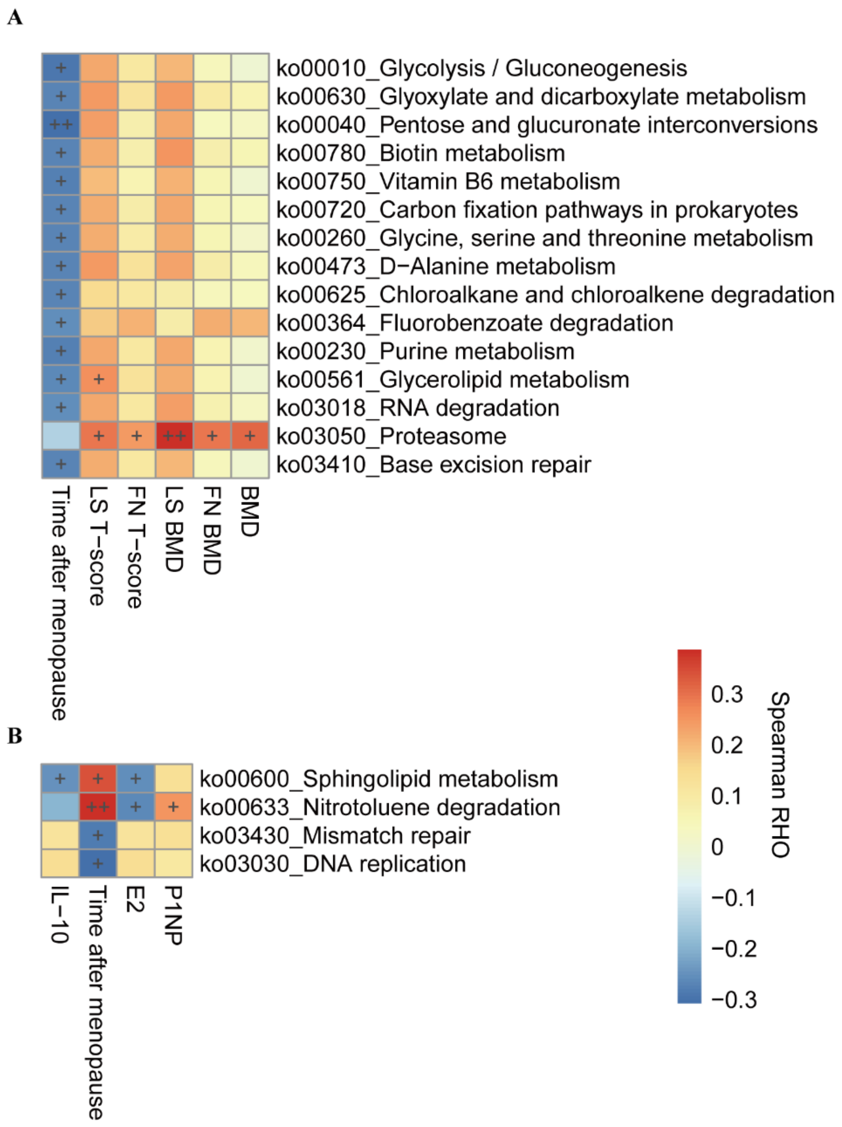

Supplementary Figure 6. Correlation between key predicted pathways and clinical parameters. (A) Correlation between key predicted pathways of GM and clinical parameters. (B) Correlation between key predicted pathways of VM and clinical parameters. MATLAB R2019b was used to calculate Spearman correlation coefficients. The Benjamini and Hochberg method was used to calculate false discovery rate (FDR) to adjust the significance of correlations. + FDR < 0.1, ++FDR < 0.01. RHO > 0 respresents postive correlation, RHO < 0 represents negative correlation.

2 Supplementary Tables

Supplementary Table 1. Clinical characteristics.

	Normal bone mass (control) group (n = 51)	Postmenopausal osteopenia group (n = 47)	Postmenopausal osteoporosis group (n = 34)	*p*
**Age (years)**	56.00 (54.00, 58.25)	58.00 (55.00, 62.00) [a]	57.50 (54.75, 63.00)	0.082
**Time after menopause (years)**	5.00 (2.00, 8.00)	7.00 (4.00, 11.00) [a]	8.00 (3.50, 13.00)	0.043*
**BMI (kg/m^2)**	23.83 (22.83, 26.44)	23.31 (21.88, 25.63)	22.11 (20.39, 24.97) [a]	0.039*
**WHR**	0.83 (0.86, 0.89)	0.87 (0.82, 0.89)	0.86 (0.81, 0.91)	0.980
**ALT (U/L)**	18.40 (15.20, 26.55)	17.20 (14.50, 22.30)	17.15 (14.43, 27.82)	0.536
**AST (U/L)**	21.90 (19.90, 26.95)	23.00 (20.20, 26.60)	22.35 (19.90, 25.98)	0.918
**TC (mmol/L)**	5.24 (4.61, 5.64)	5.26 (4.53, 5.74)	5.03 (4.63, 5.84)	0.941
**TG (mmol/L)**	1.25 (0.88, 1.92)	1.19 (0.98, 1.53)	1.13 (0.94, 1.72)	0.736
**HDL (mmol/L)**	1.39 (1.17, 1.65)	1.42 (1.28, 1.66)	1.57 (1.33, 1.71)	0.176
**LDL (mmol/L)**	3.16 (2.66, 3.58)	3.23 (2.58, 3.56)	3.07 (2.59, 3.68)	0.927
**E2 (pg/mL)**	22.59 (17.93, 29.91)	22.11 (15.93, 27.50)	13.89 (10.56, 26.76) [b, c]	0.019*
**25(OH)VD3 (nmol/L)**	16.72 (12.79, 23.04)	17.08 (13.59, 27.44)	21.76 (15.13, 26.36)	0.363
**Osteocalcin (ng/mL)**	22.83 (18.63, 28.21)	23.85 (18.5, 28.36)	24.75 (18.37, 27.92)	0.780
**P1NP (ng/mL)**	57.43 (43.25, 75.13)	60.87 (48.99, 67.59)	56.71 (47.62, 73.02)	0.976
**β-Crosslaps (ng/mL)**	0.49 (0.30, 0.62)	0.46 (0.36, 0.56)	0.45 (0.34, 0.59)	0.970
**Glucose (mmol/L)**	5.10 (4.70, 5.50)	5.30 (4.90, 5.60)	5.35 (5.03, 5.50)	0.443
**LS T-score**	0.00 (-0.50, 0.90)	-1.30 (-1.70, -1.00) [b]	-2.50 (-2.90, -2.40) [b, d]	<0.001**
**FN T-score**	-0.40 (-0.70, 0.20)	-1.25 (-1.72, -1.00) [b]	-1.90 (-2.57, -1.20) [b, d]	<0.001**
**LS BMD (g/cm^2)**	1.11 (1.05, 1.18)	0.95 (0.90, 0.99) [b]	0.82 (0.77, 0.85) [b, d]	<0.001**
**FN BMD (g/cm^2)**	0.90 (0.85, 0.96)	0.78 (0.72, 0.82) [b]	0.72 (0.62, 0.82) [b, c]	<0.001**
**BMD (g/cm^2)**	0.94 (0.88, 1.01)	0.82 (0.78, 0.94) [b]	0.73 (0.66, 0.80) [b, d]	<0.001**

Values are expressed as the median (25th-75th percentile). One-way ANOVA test or Kruskal−Wallis test was used to analyze differences among the three groups. * and ** indicate significant difference at $p < 0.05$ and $p < 0.01$, respectively. Mann-Whitney U test was used to detect the differences between the two groups. [a] $P < 0.05$ vs Control group, [b] $P < 0.01$ vs Control group, [c] $P < 0.05$ vs Osteopenia group, [d] $P < 0.01$ vs Osteopenia group. 25(OH)VD3; 25-hydroxyvitamin D_3; ALT, alanine aminotransferase; AST,

aspartate aminotransferase; BMD, bone mineral density; BMI, body mass index; E2, estrogen; FN, femoral neck; HDL, high-density lipoprotein; LDL, low-density lipoprotein; LS, lumber spine; P1NP, procollagen type 1 N-terminal propeptide; TC, total cholesterol; TG, triglyceride; WHR, waist-hip ratio.

Supplementary Table 2. Analysis of adjusted covariates of GM

Gut microbiome PerMANOVA result											Gut microbiome Anova CCA result			
Compare groups	Unadjusted P Statistics of Test		Statistics of Test Adjusted with covariate								Statistics of Test Adjusted with covariates			
			Time after menopause		Age		E2		BMI		Time after menopause +Age +BMI+E2		Time after menopause+BMI+E2	
	P	***F***	***P***	***F***	***P***	***F***	***P***	***F***	***P***	***F***	***P***	***F***	***P***	***F***
**Control vs Osteopenia**	0.021*	1.831	0.017*	2.162	0.060	2.585	0.077	2.559	0.018*	2.585	0.048*	1.134	0.640	0.969
**Control vs Osteoporosis**	0.030*	1.636	0.017*	1.538	0.027*	2.223	0.064	2.961	0.012*	2.223	0.123	1.096	0.086	1.130
**Osteopenia vs Osteoporosis**	0.010*	2.125	0.006**	1.394	0.008**	1.322	0.025*	1.246	0.023*	1.322	0.039*	1.158	0.041*	1.156

PerMANOVA and Anova CCA test were both performed by R package vegan. * and ** indicate significant difference at $p < 0.05$ and $p < 0.01$, respectively.

Supplementary Table 3. Analysis of adjusted covariates of VM

Vaginal microbiome PerMANOVA result											Vaginal microbiome Anova CCA result			
Compare groups	Unadjusted Statistics of Test		Statistics of Test Adjusted with covariate								Statistics of Test Adjusted with covariates			
			Time after menopause		Age		E2		BMI		Time after menopause +Age +BMI+E2		Time after menopause+BMI+E2	
	P	***F***	***P***	***F***	***P***	***F***	***P***	***F***	***P***	***F***	***P***	***F***	***P***	***F***
**Control vs Osteopenia**	0.006**	2.815	0.029*	1.816	0.008**	1.800	0.033*	1.514	0.018*	1.800	0.027*	1.266	0.008**	1.399
**Control vs Osteoporosis**	0.024*	2.302	0.094	1.767	0.091	1.834	0.383	2.129	0.022*	1.834	0.013*	1.249	0.003**	1.375
**Osteopenia vs Osteoporosis**	0.174	1.302	0.128	2.026	0.067	1.865	0.211	1.773	0.172	1.865	0.200	1.084	0.129	1.138

PerMANOVA and Anova CCA test were both performed by R package vegan. * and ** indicate significant difference at $p < 0.05$ and $p < 0.01$, respectively.

Supplementary Table 4. Analysis of intergroup differences in IL-33 and 17A levels

Compare groups	IL-33		IL-17A	
	P	*Z*	*P*	*Z*
Control vs Osteopenia	0.758	-0.308	0.595	-0.534
Control vs Osteoporosis	0.236	-1.184	0.328	-0.979
Osteopenia vs Osteoporosis	0.576	-0.560	0.152	-1.434

Mann-Whitney U test was used to detect the differences between the two groups. IL-33: interleukin-33; IL-17A: interleukin-17A.

Supplementary Table 5. Analysis of adjusted covariates of serum level inflammatory

	***P* value Adjusted with covariate**												***P* value Adjusted with covariates**					
	**Time after menopause**			**Age**			**E2**			**BMI**			**Time after menopause +Age +BMI+E2**			**Time after menopause+BMI+E2**		
	P_1	P_2	P_3	P_1’	P_2’	P_3’	P_1’’	P_2’’	P_3’’	P_1’’’	P_2’’’	P_3’’’	$P_1^{\#}$	$P_2^{\#}$	$P_3^{\#}$	$P_1^{\#\#}$	$P_2^{\#\#}$	$P_3^{\#\#}$
**IL-10**	0.845	0.095	0.023*	0.685	0.063	0.030*	0.427	0.070	0.095	0.549	0.076	0.019*	0.561	0.91	0.009**	0.892	0.229	0.177
**TNF-α**	0.015*	0.015*	0.695	0.013*	0.020*	0.651	0.025*	0.006**	0.501	0.028*	0.013*	0.775	0.501	0.893	0.611	0.016*	0.002**	0.203
**LBP**	0.097	0.082	0.121	0.175	0.117	0.132	0.236	0.113	0.143	0.432	0.122	0.147	0.097	0.724	0.994	0.128	0.070	0.127

All the covariates were adjusted using analysis of covariance (ANCOVA) by R package multcomp. * and ** indicate significant difference at $p < 0.05$ and $p < 0.01$, respectively. P_1 indicates *P* between the control group and osteopenia group; P_2 indicates *P* between the control group and osteoporosis group; P_3 indicates *P* between the osteopenia group and osteoporosis group.

	***F* value Adjusted with covariate**												***F* value Adjusted with covariates**					
	**Time after menopause**			**Age**			**E2**			**BMI**			**Time after menopause Age+BMI+E2+**			**Time after menopause+BMI+E2**		
	F_1	F_2	F_3	F_1’	F_2’	F_3’	F_1’’	F_2’’	F_3’’	F_1’’’	F_2’’’	F_3’’’	$F_1^{\#}$	$F_2^{\#}$	$F_3^{\#}$	$F_1^{\#\#}$	$F_2^{\#\#}$	$F_3^{\#\#}$
**IL-10**	0.039	2.892	5.478	0.166	3.613	4.966	0.640	3.451	2.914	0.362	3.282	5.840	0.343	0.013	7.584	0.019	1.49	1.888
**TNF-α**	6.253	6.317	0.156	6.518	5.801	0.207	5.335	8.346	0.460	5.049	6.626	0.082	0.459	0.018	0.263	6.175	11.384	1.674
**LBP**	2.845	3.153	2.482	1.88	2.543	2.337	1.435	2.613	2.225	0.626	2.481	2.166	2.861	0.127	0.000	2.395	3.465	2.432

All the covariates were adjusted using analysis of covariance (ANCOVA) by R package multcomp. F_1 indicates *F* value between the control group and osteopenia group; F_2 indicates *F* value between the control group and osteoporosis group; F_3 indicates *F* value between the osteopenia group and osteoporosis group.

Supplementary Table 6. Comparisons of key gut genus (Supplementary Table 5A) and predicted ko (Supplementary Table 5B) (separate document).

Supplementary Table 7. Comparisons of key vaginal genus (Supplementary Table 6A) and predicted ko (Supplementary Table 6B) (separate document).

Supplementary Table 8. Relative abundance of gut microbiota according to genus in the three cohorts (separate document).

Supplementary Table 9. Relative abundance of vaginal microbiota according to genus in the three cohorts (separate document).

Supplementary Table 10. Significant Spearman correlations between vaginal and gut microbiota according to genus (separate document).

Supplementary Table 11. Significant Spearman correlations between the genera of vaginal microbiota and clinical parameters (separate document).

Supplementary Table 12. Significant Spearman correlations between the genera of gut microbiota and clinical parameters (separate document).
